# Effect of short-term intensive insulin pump therapy on serum endotrophin levels in patients with newly diagnosed type 2 diabetes

**DOI:** 10.3389/fendo.2025.1658272

**Published:** 2025-10-08

**Authors:** Yuting Li, Xiaoting Gui, Yu Weng, Lizhang Xun, Weinan Yu, Chunli Xiang, Xing Qi, Xiaoqing Wang, Feng Bai

**Affiliations:** ^1^ Department of Endocrinology, The Huai’an Hospital Affiliated to Xuzhou Medical University and The Second People’s Hospital of Huai’an, Xuzhou Medical University, Huai’an, China; ^2^ Department of General Medicine, The Huai’an Hospital Affiliated to Xuzhou Medical University and The Second People’s Hospital of Huai’an, Xuzhou Medical University, Huai’an, China; ^3^ Medical Examination Center, The Huai’an Hospital Affiliated to Xuzhou Medical University and The Second People’s Hospital of Huai’an, Xuzhou Medical University, Huai’an, China

**Keywords:** endotrophin, type 2 diabetes, short-term intensive insulin pump therapy, insulin resistance, inflammation

## Abstract

**Objective:**

To investigate the effect of short-term intensive insulin (STII) pump therapy on serum endotrophin (ETP) levels in patients with newly diagnosed type 2 diabetes, examine the relationship between serum ETP levels and insulin resistance (IR).

**Subjects and methods:**

Our study recruited 40 patients with newly diagnosed type 2 diabetes and 40 healthy controls. First, we compared the serum ETP levels between patients with newly diagnosed type 2 diabetes and controls. Second, 40 patients with newly diagnosed type 2 diabetes underwent two weeks of STII pump therapy.

**Results:**

Higher serum ETP levels were observed in patients with newly diagnosed type 2 diabetes compared to controls. After 2 weeks of STII pump therapy, serum ETP levels significantly attenuated in patients with newly diagnosed type 2 diabetes. Both before and after treatment, serum ETP exhibited a significant positive correlation with homeostasis model 2 assessment of IR (HOMA2-IR). Multiple linear regression analysis revealed that changes in ETP levels (δ-ETP) were independently associated with changes in HOMA2-IR (δ-HOMA2-IR).

**Conclusions:**

Serum ETP serves as a risk factor for type 2 diabetes and correlates with insulin resistance index. STII contributed to reducing the heightened inflammatory response in individuals with type 2 diabetes.

## 1 Introduction

Diabetes stands as one of the most prevalent and serious chronic diseases worldwide. Currently, more than 537 million people worldwide are affected by diabetes. Estimates suggest that the total number of diabetes patients will rise to 784 million by 2045. Among all countries, China has the largest diabetic population (1). Substantial evidence indicates that sustained hyperglycemia may induce a range of diabetes-related complications, particularly nephropathy and retinopathy. Inflammatory processes are implicated as a critical factor in the onset and progression of these conditions (2).

Chronic low-grade inflammation in adipose tissue connects obesity-related insulin resistance (IR) to type 2 diabetes. Numerous studies have demonstrated that adipose tissue serves not only as a critical organ for regulating energy storage and nutrient balance but also as an endocrine organ capable of secreting various adipokines involved in glucose and lipid metabolism regulation (3, 4). Collagen VI (COL6) is one of the extracellular matrix proteins secreted by adipose tissue, playing a crucial role in maintaining the structural integrity and functionality of various tissues throughout the body. Endotrophin (ETP), a signaling fragment generated during COL6 formation (5), has been shown to exhibit pro-inflammatory and pro-fibrotic properties, contributing to systemic IR and adverse metabolic outcomes, such as lipid toxicity (6–8). Recent mouse studies indicated that induced overexpression of ETP in adipocytes exacerbates IR, whereas treatment with ETP-neutralizing antibodies enhances insulin sensitivity and alleviates adipose tissue inflammation and fibrosis in obese mice. Further human studies revealed elevated serum ETP levels in patients with IR (9–11).

Short-term intensive insulin (STII) therapy has increasingly become a first-line treatment for newly diagnosed type 2 diabetes patients in recent years (12). This approach achieves non-pharmacological remission by rapidly and effectively normalizing blood glucose levels. The primary mechanism of STII involves reducing glucotoxicity and relieving the excessive workload on beta cells, which enhances metabolic control and preserves pancreatic islet function (13, 14). A hyperglycemic environment is associated with chronic inflammation, which exacerbates IR and may induce oxidative stress, leading to further damage to blood vessels and other tissues (2). By lowering blood glucose levels efficiently, STII therapy reduces inflammatory factor release, improves insulin sensitivity, and inhibits hyperglycemia-induced oxidative stress (15).Therefore, we hypothesized that STII treatment might improve the degree of IR by modulating the levels of the proinflammatory adipokine ETP.

However, no studies have specifically examined the relationship between serum ETP and newly diagnosed type 2 diabetes. Moreover, the impact of STII on ETP levels remains unexplored. In this study, we aim to investigate the effects of STII on ETP levels in newly diagnosed type 2 diabetes. Additionally, we demonstrated the anti-inflammatory effects of STII by comparing changes in ETP levels before and after STII therapy in patients with newly diagnosed type 2 diabetes.

## 2 Materials and methods

### 2.1 Subjects

Our study enrolled 40 patients with newly diagnosed type 2 diabetes who received STII pump therapy at the Department of Endocrinology, Huai’an Hospital Affiliated to Xuzhou Medical University. The inclusion criteria were as follows: (1) aged 18–70 years, meeting the WHO 1999 diagnostic criteria for diabetes, and not having previously received any hypoglycemic medications; (2) having an HbA1c level of 9.0% (75 mmol/mol) or higher, which according to the Chinese Guidelines for the Prevention and Management of type 2 diabetes (2020 edition) (16), indicates the need for intensive insulin therapy; (3) being willing to comply with dietary and exercise recommendations. The exclusion criteria included: (1) individuals with type 1 diabetes mellitus, gestational diabetes, or other special types of diabetes; (2) those with acute infections or diabetic complications such as diabetic nonketotic hyperosmolar coma or diabetic ketoacidosis; (3) patients suffering from severe cardiovascular, hepatic, renal, or cerebrovascular diseases; (4) individuals who had used systemic corticosteroids within the three months prior to enrollment; (5) those with a history of malignant diseases; (6) participants with a history of pancreatectomy or acute/chronic pancreatitis.

Additionally, 40 healthy volunteers were recruited from the Health Examination Center of Huaian Hospital Affiliated to Xuzhou Medical University. The inclusion criteria for the control group were as follows: (1) aged 18–70 years, Fasting venous blood glucose between 3.9 mmol/L and 6.1 mmol/L, and a 2-hour postprandial venous blood glucose level below 7.8 mmol/L following an oral glucose tolerance test; (2) Glycated hemoglobin (HbA1c) below 6.1%. (3) Individuals with no history of diabetes or major chronic diseases. The exclusion criteria were consistent with those applied to the type 2 diabetes group. All participants provided written informed consent prior to the commencement of the study.

### 2.2 Study design and sample collection

Upon admission, baseline parameters such as height, weight, age, and medical history were documented. Body mass index (BMI) was calculated as weight (kg) divided by height squared (m²). Following an 8-hour overnight fast, blood samples were obtained from all participants to measure fasting plasma glucose (FPG), fasting C-peptide (FC-P), blood urea nitrogen (BUN), uric acid (UA), creatinine (CR), triglycerides (TG), total cholesterol (TC), low-density lipoprotein cholesterol (LDL-C), high-density lipoprotein cholesterol (HDL-C), and glycated hemoglobin (HbA1c) using an integrated automated analyzer (Roche, China). Serum ETP levels were quantified using a human ETP-specific enzyme-linked immunosorbent assay kit (Jiangsu Meimian Industrial Co Ltd, China). The estimated glomerular filtration rate (eGFR) was determined using the chronic kidney disease epidemiology collaboration (CKD-EPI) equation (17). Fasting blood glucose and fasting C-peptide values were input into the software (https://www.ox.ac.uk), and the homeostatic model assessment (HOMA) was then computed (18).

All patients with newly diagnosed type 2 diabetes underwent hospitalization for STII pump therapy. All participants hospitalized for STII therapy received standardized dietary guidance and were encouraged to maintain light daily physical activity, following institutional protocols. The initial insulin dose was established at 0.5‐0.8 IU/kg/day, with half of the total daily dose administered as basal insulin over a 24‐hour period and the remaining half evenly distributed before each of the three meals. Insulin infusion protocols were adjusted according to capillary blood glucose levels, which were measured seven times daily (upon waking, preprandially, and 2 hours postprandially). Glycemic control objectives during the first 5‐7 days of STII therapy included maintaining fasting plasma glucose (FPG) below 7.0 mmol/L and 2‐hour postprandial glucose (2hPG) below 10 mmol/L, followed by maintenance therapy for a total of 2 weeks. Blood samples obtained prior to and following intensive insulin therapy were centrifuged at 3000 rpm for 20 minutes, and the resulting supernatant was separated and stored at −80 °C. All stored blood samples were thawed only once on the day of analysis.

### 2.3 Statistical analysis

Statistical analysis was conducted using SPSS 27.0 and GraphPad Prism 9. Normally distributed variables are presented as mean ± standard deviation (SD), whereas non-normally distributed variables are expressed as median (interquartile range, IQR). Categorical data are summarized as frequencies or percentages. For comparisons of continuous variables between two groups, the t-test was used for normally distributed data, while the Mann–Whitney U-test was applied for non-normally distributed data. When comparing more than two groups, one-way ANOVA with *post hoc* Bonferroni correction was performed for normally distributed data, and the Kruskal–Wallis H-test with Dunn’s multiple comparison test was used for non-normally distributed data. The association between ETP and type 2 diabetes was evaluated using a logistic regression model. Spearman rank correlation analysis was utilized to examine the correlation between serum ETP and other clinical parameters. For each variable, the difference δ was calculated as the initial value minus the value at 2 weeks (δ = old value - new value). Factors associated with δ-ETP levels were further analyzed using multiple linear regression to identify independent predictors of δ-ETP changes. In all statistical analyses, two-tailed *P* values were reported, and *P* < 0.05 was considered statistically significant.

## 3 Results

### 3.1 Clinical and biochemical characteristics at baseline

A total of 80 subjects were enrolled in this study, including 40 patients with newly diagnosed type 2 diabetes and 40 healthy controls. The baseline clinical characteristics of the participants are presented in **Table 1**. The mean age (± SD) was 45.30 ± 12.53 years in the T2D group and 45.10 ± 12.83 years in the control group. Compared with the control group, patients with newly diagnosed type 2 diabetes exhibited significantly higher levels of BMI, CRP, FPG, HbA1c, HOMA2-IR, and TG (*P* < 0.05). In contrast, DBP, HOMA2-β, CR, UA, and HDL-C levels were markedly reduced in this group (*P* < 0.05). These findings are consistent with previous studies, which have demonstrated that patients with newly diagnosed type 2 diabetes typically present with lower HOMA2-β but higher HOMA2-IR (*P* < 0.05), suggesting the presence of IR and a decline in β-cell function.

**Table 1:** Characteristics of all participants.

Indicator	Control (n =40)	T2DM (n =40)	*P* value
Gender (Male,%)	31(77.5%)	31(77.5%)	1.000
Age (years)	45.30 ± 12.53	45.10 ± 12.83	0.801
Height (m)	1.70 ± 0.07	1.69 ± 0.07	0.642
Body weight (kg)	71.9 ± 9.64	76.46 ± 13.11	0.179
BMI (kg/m^2^)	24.82 ± 2.72	26.6 ± 3.78	0.006*
SBP (mmHg)	129.50(114.50,136.75)	125.50(122.00,130.00)	0.992
DBP (mmHg)	82.00(74.00,88.00)	76.00(70.00,78.00)	0.023*
CRP (mg/L)	0.55(0.25,1.01)	2.50(0.55,4.52)	<0.001*
FPG (mmol/L)	4.92(4.76,5.49)	13.45(10.70,14.46)	<0.001*
FC-P (ng/ml)	2.10(1.71,2.58)	2.48(1.77,3.46)	0.156
FINS (pmol/L)	57.30(36.15,76.68)	63.30(41.30,114.25)	0.197
HOMA2-IR	1.52 (1.291.95)	2.56(1.64,4.19)	<0.001*
HOMA2-β	126.75(103.00,162.80)	28.35(16.90,43.90)	<0.001*
HbA1c (%)	5.68 ± 0.22	11.17 ± 1.61	<0.001*
BUN (mmol/L)	5.27(3.72,6.01)	5.27(4.23,6.15)	0.551
CR (μmol/L)	60.00(56.00,67.00)	53.00(44.25,67.35)	0.029*
eGFR (ml/min/1.73 m^2^)	110.36 ± 12.65	117.17 ± 19.97	0.073
UA (μ mol/L)	346.55 ± 74.368	327.15 ± 106.70	0.023*
TC (mmol/L)	4.68(4.16,5.01)	5.11(4.13,5.87)	0.135
TG (mmol/L)	1.68(1.03,2.36)	2.14(1.44,4.14)	0.023*
HDL-C (mmol/L)	1.23(1.06,1.35)	1.00(0.83,1.21)	<0.001*
LDL-C (mmol/L)	2.96(2.35,3.43)	2.75(2.16,3.70)	0.912
ETP (pg/ml)	236.64(223.80,248.22)	276.47(270.14,284.09)	<0.001*

Continuous parametric variables were reported as means ± SD. Continuous nonparametric variables were described as medians (interquartile ranges), and categorical variables were expressed as proportions.

BMI, body mass index; SBP, systolic blood pressure; DBP, diastolic blood pressure; CRP, C-reactive protein; FPG, fasting plasma glucose; FC-P, fasting C-peptide; FINS, fasting insulin; HbA1c, glycated hemoglobin; HOMA2-IR, homeostasis model 2 assessment of IR; HOMA2-β, Homeostasis model 2 assessment-β; BUN, urea nitrogen; CR, serum creatinine; eGFR, estimated glomerular filtration rate; UA, uric acid; TC, total cholesterol; TG, triacylglycerol; HDL-C, high-density lipoprotein cholesterol; LDL-C, low-density lipoprotein cholesterol; ETP, endotrophin. *p < 0.05, indicating statistical significance.

### 3.2 Relationship between serum ETP and newly diagnosed type 2 diabetes

To explore the relationship between serum ETP and newly diagnosed type 2 diabetes, a logistic regression analysis was performed. Univariate analysis identified BMI, CRP, HOMA2-IR, HDL-C, and ETP as significantly associated factors with type 2 diabetes (P < 0.05, data not shown). Binary logistic regression further demonstrated that serum ETP levels (OR = 1.308, 95% CI: 1.125–1.521, *P* = 0.001) were positively correlated with the presence of type 2 diabetes (**Table 2**). These results suggest that elevated serum ETP may act a an risk factor for newly diagnosed type 2 diabetes.

**Table 2:** Odds ratios for associations between targets and newly diagnosed type 2 diabetes.

Indicator	β	OR	*P*	95%CI
ETP (pg/ml)	0.269	1.308	<0.001*	(1.125,1.521)
BMI (kg/m^2^)	0.094	1.127	0.204	(0.937,1.355)
CRP (mg/L)	0.251	1.862	0.013*	(1.139,3.044)
HOMA2-IR	0.349	2.204	0.024*	(1.112,4.367)
HDL-C (mmol/L)	1.078	0.700	0.741	(0.085,5.792)

ETP, endotrophin; BMI, body mass index; HDL-C, high-density lipoprotein cholesterol; HOMA2-IR, homeostasis model 2 assessment of IR; CRP, C-reactive protein. *p < 0.05, indicating statistical significance.

### 3.3 ETP serum level

In this study, serum ETP concentration was significantly higher in the newly diagnosed diabetic group than in the control group (*P* < 0.001, **Table 1**). After two weeks of STII therapy, serum ETP levels decreased markedly compared to baseline values, changing from 276.47 (270.14, 284.09) pg/mL to 249.51 (243.61, 263.58) pg/mL (*P* < 0.001, **Table 3**). Furthermore, following two weeks of STII therapy, significant reductions were observed in serum levels of CRP, FPG, FC-P, HOMA2-IR, UA, TC, TG, and LDL-C compared to pre-treatment levels, while HOMA2-β showed significant increases (**Table 3**, *P* < 0.05).

**Table 3:** Changes in indices in the diabetic group before and after intensive insulin therapy.

Indicator	Pre-treatment	Post-treatment	z	*P*
SBP (mmHg)	125.50(122.00,130.00)	123.50(120.00,125.7)	-2.021	0.053
DBP (mmHg)	75.00(70.00,78.00)	75.00(70.00,76.00)	-0.929	0.353
CRP (mg/L)	2.50(0.55,4.52)	0.21(0.20,1.88)	-4.959	<0.001*
FPG (mmol/L)	13.45(10.70,15.46)	6.07(5.57,6.25)	-5.511	<0.001*
FC-P (ng/ml)	2.48(1.77,3.46)	2.02(1.44,2.92)	-2.635	0.008
HOMA2-IR	2.56(1.64,4.19)	1.52(1.11,2.27)	-4.597	<0.001*
HOMA2-β	28.35(16.9,43.90)	90.15(69.30,119.68)	5.511	<0.001*
BUN (mmol/L)	5.27(4.23,6.15)	4.73(4.05,5.68)	-1.389	0.165
CR (μ mol/L)	53.00(44.25,67.35)	62.00(44.50,67.50)	-2.119	0.054
eGFR (ml/min/1.7m^2^)	118.85(103.58,132.48)	113.00(105.85,119.63)	-1.063	0.288
UA (μmol/L)	322.00(230.25,388.50)	285.00(216.00,344.50)	-2.442	0.015*
TC (mmol/L)	5.11(4.13,5.87)	3.71(3.34,4.83)	-4.654	<0.001*
TG (mmol/L)	2.14(1.44,4.14)	1.48(1.05,2.27)	-3.740	<0.001*
HDL-C (mmol/L)	1.00(0.83,1.21)	1.03(0.87,1.26)	-1.079	0.281
LDL-C (mmol/L)	2.75(2.16,3.70)	2.16(1.72,2.92)	-2.931	0.003*
ETP (pg/ml)	276.47(270.14,284.09)	249.51(243.61,263.58)	-5.403	<0.001*

SBP, systolic blood pressure; DBP, diastolic blood pressure; CRP, C-reactive protein; FPG, fasting plasma glucose; FC-P, fasting C-peptide; HOMA2-IR, homeostasis model 2 assessment of IR; HOMA2-β, Homeostasis model 2 assessment-β; BUN, urea nitrogen; CR, serum creatinine; eGFR, estimated glomerular filtration rate; UA, uric acid; TC, total cholesterol; TG, triacylglycerol; HDL-C, high-density lipoprotein cholesterol; LDL-C, low-density lipoprotein cholesterol; ETP, endotrophin. *p < 0.05, indicating statistical significance.

### 3.4 Relationship analysis

**Figure 1:**
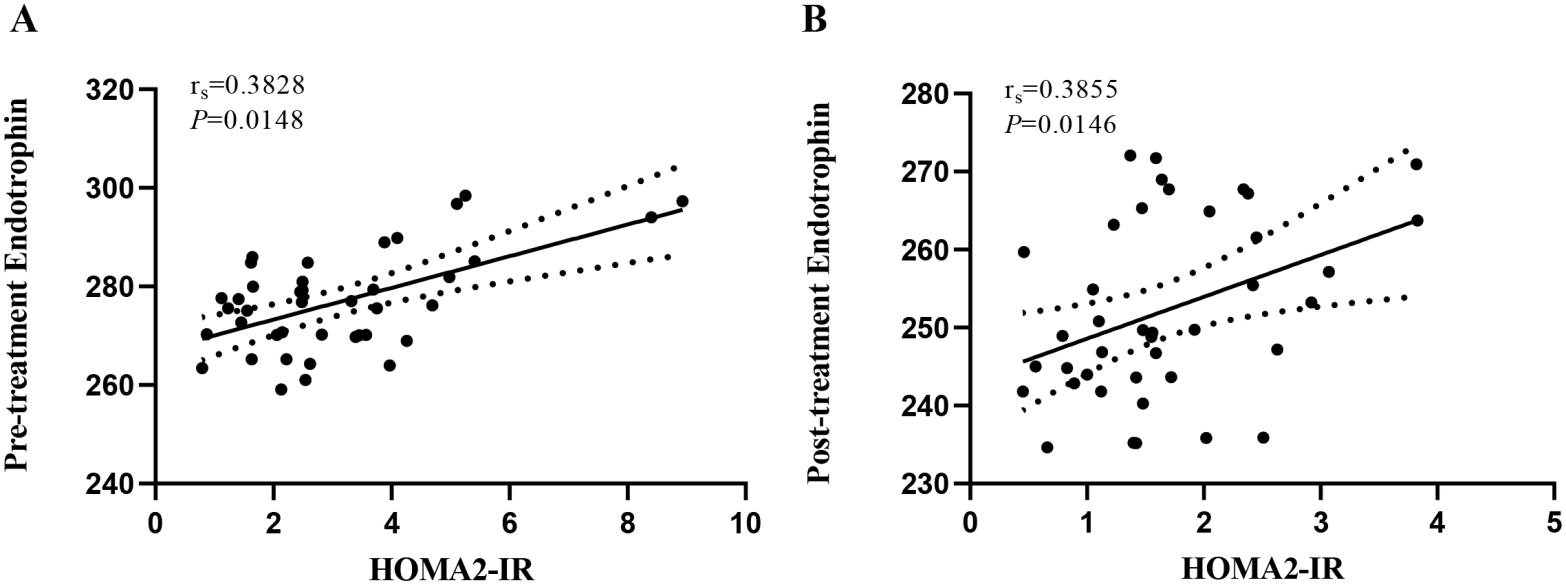
Correlation between serum ETP level and HOMA2-IR before and after insulin intensive therapy. HOMA2-IR, homeostasis model 2 assessment of IR; ETP, endotrophin. **(A)** reveals the correlation between serum ETP levels and HOMA2-IR in pre-treatment patients with newly diagnosed type 2 diabetes (rs = 0.3828, P = 0.0148). **(B)** reveals the correlation between serum ETP levels and HOMA2-IR in post-treatment patients with newly diagnosed type 2 diabetes (rs = 0.3855, P = 0.0146).

In this study, we first investigated the association between serum ETP and the IR index in patients with newly diagnosed type 2 diabetes before and after intensive insulin therapy. The detailed results of the Spearman correlation analysis are presented in **Figure 1**. Prior to STII pump therapy, the level of ETP exhibited a moderate positive correlation with HOMA2-IR (r=0.3828, *P* = 0.0148). Following two weeks of STII pump therapy, serum ETP remained moderately positively correlated with HOMA2-IR (r=0.3855, *P* = 0.0146).

Multiple linear regression analysis revealed that δ-ETP levels were independently associated with δ-HOMA2-IR (**Table 4**, model r^2^ = 0.512, *P* = 0.02). Furthermore, the change in FPG did not exhibit a significant relationship with the change in serum ETP levels (*P* > 0.05, data not shown).

**Table 4:** Multiple linear regression analysis of delta-ETP as dependent variable.

Independent variable	B	SE	β	T value	*P* value
Constant	14.518	7.190	–	2.019	0.054
δ-HOMA2-IR	-0.475	0.139	-0.915	-3.407	0.002
δ-UA	0.066	0.023	0.538	2, 817	0.009

δ-ETP =14.518-0.475*δ-HOMA2-IR+0.066*δ-UA

### 3.5 The incidence of hypoglycemia

Hypoglycemia was defined as blood glucose < 3.9 mmol/L accompanied by symptoms such as hunger, trembling, anxiety, sweating, dizziness, or lightheadedness. During the 2-week STII treatment, two patients with newly diagnosed type 2 diabetes experienced hypoglycemic events. None of these episodes required intervention with intravenous glucose or glucagon. Insulin doses were promptly adjusted, and no recurrent hypoglycemia occurred in any patient.

## 4 Discussion

In this study, we observed a significant elevated in serum ETP levels in patients with newly diagnosed type 2 diabetes, indicating a positive association between serum ETP and type 2 diabetes. Tougaard NH et al. also reported that serum COL6 formation marker ETP was independently associated with renal events, first major adverse cardiovascular events, mortality, and the risk of heart failure in patients with type 2 diabetes. Additionally, urinary ETP was found to be associated with the progression of proteinuria (19). These fundings may support a hypothesis that serum ETP plays a significant pathogenic role in the initiation and progression of type 2 diabetes.

The novel adipokine ETP, a signaling peptide derived from the cleavage of the COL6 α3 chain, has emerged as a critical mediator in the pathological processes of adipose tissue in obesity. Evidence linking elevated ETP levels in obese adipose tissue to diabetes development suggested that ETP not only contributes to an adverse microenvironment within obese adipose tissue but also serves as a pathologically relevant driving force. This ultimately results in systemic IR and obesity-related metabolic disorders (6, 20). Elevated expression levels of ETP promote macrophage infiltration into adipose tissue, which is a major source of circulating inflammatory factors in obesity and plays a critical role in the progression of IR. Moreover, adipose tissue with increased ETP levels rapidly disrupts the regulation of circulating TG and non-esterified fatty acids, leading to hepatic steatosis. Consequently, abnormal lipid accumulation occurs in other tissues or organs, creating a “lipid toxic” environment that further exacerbates IR (20–22).

Preclinical studies have revealed that overexpression of ETP in the adipose tissue of transgenic mice impairs normal adipocyte function, leading to systemic metabolic dysfunction, characterized by enhanced IR and reduced insulin sensitivity (6). Additionally, transgenic mice exhibited significantly elevated levels of transforming growth factor-β1 (TGF-β1) and its receptors in adipose tissue (23). Increased oxygen radical production due to glucotoxicity has been shown to stimulate TGF-β synthesis. Similar to glucotoxicity, ETP exerts pleiotropic effects by potentiating TGF-β synthesis, thereby positively regulating diverse biological processes including inflammation, angiogenesis, fibrosis, and epithelial-mesenchymal transition (24, 25). At high glucose concentrations, fibrous structures form within the extracellular matrix surrounding adipose tissue, damaging the microenvironment and inducing elevated COL6 levels. However, regulating blood glucose can inhibit macrophage and fibroblast activity, potentially reducing ETP concentrations during effective glucose control (26). Sengul et al. observed that circulating ETP levels decreased alongside reductions in glycated hemoglobin and blood glucose, suggesting that improved glycemic control may lead to lower ETP concentrations (45). Our study demonstrated a positive correlation between serum ETP and fasting blood glucose before treatment in newly diagnosed type 2 diabetic patients. However, no significant correlation was found between serum ETP and fasting blood glucose after two weeks of STII therapy, nor was there a correlation between the changes in ETP and the changes in fasting blood glucose before and after treatment. Both glucotoxicity and ETP can modulate metabolic processes by enhancing TGF-β synthesis. However, current evidence remains insufficient to elucidate the interaction between glucose and ETP. The underlying signaling pathways and mechanisms require further investigation.

This study examined the changes in serum ETP levels in patients with newly diagnosed type 2 diabetes following STII pump therapy. Our results revealed that serum ETP levels in patients with newly diagnosed type 2 diabetes, which were markedly reduced following STII pump therapy. Additionally, we observed a positive correlation between baseline serum ETP levels and HOMA2-IR in patients with newly diagnosed type 2 diabetes. Furthermore, the reduction in ETP levels following STII pump therapy was also positively correlated with HOMA2-IR, suggesting that ETP may be associated with IR. Multiple linear regression analysis demonstrated that the δ-HOMA2-IR value could effectively explain variations in δ-ETP levels. This implies that larger changes in HOMA2-IR are associated with more pronounced fluctuations in ETP levels, while smaller changes in HOMA2-IR correspond to minimal alterations in ETP levels among in patients with type 2 diabetes. A recent study revealed that circulating ETP levels are associated with insulin resistance in individuals with obesity (44). Fasting ETP was significantly higher in obese insulin-resistant subjects compared to obese insulin-sensitive individuals. Using dietary induction, it was found that the 24-hour area under the curve (AUC) for circulating ETP was 25% higher in obese insulin-resistant subjects than in obese insulin-sensitive controls. In obese patients with type 2 diabetes, significant weight loss achieved through surgical intervention or intensive dietary therapy led to a marked reduction in circulating ETP levels alongside improved insulin sensitivity. Furthermore, endotrophin has been shown to directly impair insulin-stimulated glucose uptake in human skeletal muscle myotubes, indicating a direct negative effect on insulin action. In our study, newly diagnosed type 2 diabetic patients were stratified by BMI, only three cases were classified into the obese group, and no significant relationship was observed between BMI and circulating ETP—a finding that may be attributed to the small sample size. Research by Samuel Klein’s team suggests that circulating ETP acts as both a biomarker and a causative factor of systemic insulin resistance in obese insulin-resistant populations. However, our preliminary findings indicate a strong correlation between circulating ETP and insulin resistance in newly diagnosed type 2 diabetic patients, but do not establish a causal relationship. Whether ETP directly influences insulin resistance in type 2 diabetes remains unclear, and further investigation is needed to elucidate its role and underlying mechanisms.

ETP promotes the synthesis of TGF-β. Pancreatic β-cells dysfunction or depletion constitutes a pivotal pathophysiological event in the development of diabetes mellitus. According to current evidence, TGF-β signaling is active in both developing and adult β cells, generally inhibiting β cell proliferation (25). In addition, high glucose contributes to the occurrence and progression of diabetic complications by upregulating the TGF-β signaling pathway (27). Therefore, studies investigating potential antidiabetic therapies targeting the TGF-β signaling pathway are becoming increasingly significant. Given this, the role of ETP in enhancing TGF-β synthesis warrants further exploration regarding its impact on diabetes and associated complications. Sun et al. found that in mice on a high-fat diet, ETP overexpression may mediate chronic inflammation and fibrosis by activating the TGF-β pathway, which plays a key role in insulin resistance. This suggests that activation of the TGF-β pathway is an integral component of ETP’s effects. Subsequently, neutralizing antibodies against ETP were shown to ameliorate adverse metabolic effects and effectively reverse metabolic dysfunction induced during high-fat diet exposure (20). Based on these findings, targeting ETP neutralization or blocking the ETP-enhanced TGF-β synthesis pathway may offer a novel strategic direction for developing new therapies for type 2 diabetes.

Type 2 diabetes represents a complex metabolic disorder characterized by two fundamental abnormalities: impaired pancreatic hormone secretion and reduced insulin sensitivity in target tissues. In adipose tissue, IR promotes lipolysis, resulting in elevated circulating free fatty acids. This not only exacerbates insulin dysfunction but also contributes to systemic inflammation, creating a vicious cycle that worsens metabolic health (28). Chronic hyperglycemia triggers a cascade of pathophysiological responses, including oxidative stress, chronic low-grade inflammation, and proinflammatory cytokine release, ultimately leading to diabetic complications and multi-organ dysfunction (29). In patients with type 2 diabetes, dyslipidemia may lead to increased concentrations of circulating inflammatory cytokines (30). Existing studies have shown that IR is closely linked to a chronic low-grade inflammatory state. Multiple immune cell populations, including macrophages, T cells, and B cells - synergize with adipocyte-derived inflammatory mediators to drive the initiation and progression of IR (31). Previous studies have demonstrated that various pro-inflammatory adipokines, including Apelin, Resistin, Visfatin, and TNF-α, contribute not only to the impairment of pancreatic hormone secretion and IR (32), but also to the development of chronic diabetic complications. For example, elevated levels of these pro-inflammatory adipokines can exacerbate oxidative stress induced by hyperglycemia, resulting in endothelial dysfunction and increased vascular permeability (33, 34). In our study, the results also demonstrated that serum ETP levels were significantly elevated in patients with newly diagnosed type 2 diabetes, further suggesting the presence of increased chronic inflammation in type 2 diabetes. Targeting ETP or its associated pathways may provide novel strategies for reducing inflammation and enhancing metabolic control in type 2 diabetes.

Accumulating clinical evidence demonstrates that short-term intensive insulin (STII) therapy rapidly normalizes glycemic levels and reduces lipid accumulation, effectively alleviating glucotoxicity, lipotoxicity, and insulin resistance (IR) in patients with newly diagnosed type 2 diabetes. This, in turn, protects β-cell function and enhances overall metabolic health. However, studies examining the effects of STII treatment on ETP expression in patients with diabettes remain scarce. Thus, determining whether STII therapy can suppress ETP expression *in vivo* would offer valuable insights into its broader therapeutic potential for reducing inflammation and improving metabolic outcomes in diabetes management.

The exact mechanisms underlying the anti-inflammatory effects of insulin therapy are still not fully understood. Nonetheless, the following evidence supports this notion: 1) Insulin can activate several signaling pathways that suppress the expression of pro-inflammatory genes, including the phosphatidylinositol 3-kinase, the protein kinase B/Akt (35). By modulating these pathways, insulin plays a crucial role in maintaining the balance between metabolic homeostasis and immune responses, thereby potentially alleviating inflammation-related complications (36–39). Moreover, insulin attenuates toll-like receptor (TLR)-mediated inflammatory damage by inhibiting TLR signaling pathways (40). 2) Insulin modulates the levels of inflammatory mediators to bridge inflammation, such as by inhibiting generation and extracellular release of pro-inflammatory factors (e.g., tumor necrosis factor-α and interleukin-6) (41), and downregulating the expression of immune mediators like monocyte chemoattractant protein-1 and nuclear factor kappa B (42). 3) Insulin therapy reduces the ratio of T helper cell type 1 to T helper cell type 2 while increasing the proportion of regulatory T cells, thereby attenuating inflammatory responses (15). 4) High blood glucose levels may stimulate leukocytes to produce reactive oxygen species (ROS) and enhance oxidative stress, thereby exacerbating inflammatory conditions. Insulin administration can inhibit ROS generation and demonstrate anti-inflammatory properties (43). 5) Insulin therapy reduces IR linked to inflammation. In this study, we found that STII pump therapy improved IR in patients with newly diagnosed type 2 diabetes. Moreover, we observed that the levels of the inflammatory marker CRP decreased as glycemic control was enhanced. Along with glycemic regulation, serum ETP levels were significantly reduced, paralleled by decreased CRP levels, suggesting ETP’s beneficial modulatory effect on systemic inflammation.

This research has certain limitations. First, it was a single-institution study with a relatively limited cohort, analyzing the relevant data of only 40 patients with newly diagnosed type 2 diabetes before and after intensive insulin therapy. Second, the relatively short duration of intensive insulin therapy, focusing solely on the dynamic changes in ETP levels during the short-term remission of hyperglycemia. Existing studies have indicated that ETP may serve as a biomarker for complications in type 2 diabetes and is associated with mortality, renal complications, and cardiovascular events. Therefore, long-term follow-up in diabetic patients is of great clinical importance. further studies would be valuable to expand the sample size and enhance follow-up investigations, exploring the changes in ETP levels at 3 months, 1 year, and beyond following STII therapy in patients with newly diagnosed type 2 diabetes.

In summary, our preliminary study demonstrated that serum ETP levels were significantly higher in patients with newly diagnosed type 2 diabetes than in the control group. Insulin treatment could decrease serum ETP expression, suggesting that STII therapy may alleviate the elevated micro-inflammatory state in patients with type 2 diabetes. However, the mechanisms underlying the anti-inflammatory effects of insulin therapy warrant further investigation. Additionally, we found that reduced ETP expression was strongly correlated with improved HOMA-IR but not with changes in FPG. This finding highlights the need for larger sample sizes and long-term studies to determine whether the anti-inflammatory effects of insulin therapy are independent of its hypoglycemic effects.

## Data Availability

The original contributions presented in the study are included in the article/supplementary material. Further inquiries can be directed to the corresponding author.
